# Supplementary material for: A fault-intrusion-tolerant system and deadline-aware algorithm for scheduling scientific workflow in the cloud
Source: PeerJ Comput Sci. 2021 Nov 2;7:e747. doi: 10.7717/peerj-cs.747 (PMC8576554; doi:10.7717/peerj-cs.747)
Supplement: Supplemental Information 6 [file peerj-cs-07-747-s006.zip › Myproject1.1/docs/org/cloudbus/cloudsim/core/class-use/SimEvent.html]

Uses of Class org.cloudbus.cloudsim.core.SimEvent


JavaScript is disabled on your browser.


- Overview
- Package
- Class
- Use
- Tree
- Deprecated
- Index
- Help

- Prev
- Next

- Frames
- No Frames

- All Classes

## Uses of Class org.cloudbus.cloudsim.core.SimEvent

- Packages that use SimEvent

  | Package | Description |
  | --- | --- |
  |  |  |
  | --- | --- |
  | org.cloudbus.cloudsim |  |
  | org.cloudbus.cloudsim.core |  |
  | org.cloudbus.cloudsim.core.predicates |  |
  | org.cloudbus.cloudsim.examples |  |
  | org.cloudbus.cloudsim.network.datacenter |  |
  | org.cloudbus.cloudsim.power |  |
  | org.workflowsim |  |
- - ### Uses of SimEvent in org.cloudbus.cloudsim

    Methods in org.cloudbus.cloudsim with parameters of type SimEvent

    | Modifier and Type | Method and Description |
    | --- | --- |
    |  |  |
    | --- | --- |
    | `protected void` | Datacenter.`processCloudlet(SimEvent ev, int type)` Processes a Cloudlet based on the event type. |
    | `protected void` | DatacenterBroker.`processCloudletReturn(SimEvent ev)` Process a cloudlet return event. |
    | `protected void` | Datacenter.`processCloudletStatus(SimEvent ev)` Process the event for an User/Broker who wants to know the status of a Cloudlet. |
    | `protected void` | Datacenter.`processCloudletSubmit(SimEvent ev, boolean ack)` Processes a Cloudlet submission. |
    | `protected void` | Datacenter.`processDataAdd(SimEvent ev, boolean ack)` Process data add. |
    | `protected void` | Datacenter.`processDataDelete(SimEvent ev, boolean ack)` Process data del. |
    | `void` | Datacenter.`processEvent(SimEvent ev)` Processes events or services that are available for this PowerDatacenter. |
    | `void` | DatacenterBroker.`processEvent(SimEvent ev)` Processes events available for this Broker. |
    | `protected void` | Datacenter.`processOtherEvent(SimEvent ev)` Here all the method related to VM requests will be received and forwarded to the related method. |
    | `protected void` | DatacenterBroker.`processOtherEvent(SimEvent ev)` Overrides this method when making a new and different type of Broker. |
    | `protected void` | Datacenter.`processPingRequest(SimEvent ev)` Processes a ping request. |
    | `protected void` | DatacenterBroker.`processResourceCharacteristics(SimEvent ev)` Process the return of a request for the characteristics of a PowerDatacenter. |
    | `protected void` | DatacenterBroker.`processResourceCharacteristicsRequest(SimEvent ev)` Process a request for the characteristics of a PowerDatacenter. |
    | `protected void` | DatacenterBroker.`processVmCreate(SimEvent ev)` Process the ack received due to a request for VM creation. |
    | `protected void` | Datacenter.`processVmCreate(SimEvent ev, boolean ack)` Process the event for an User/Broker who wants to create a VM in this PowerDatacenter. |
    | `protected void` | Datacenter.`processVmDestroy(SimEvent ev, boolean ack)` Process the event for an User/Broker who wants to destroy a VM previously created in this PowerDatacenter. |
    | `protected void` | Datacenter.`processVmMigrate(SimEvent ev, boolean ack)` Process the event for an User/Broker who wants to migrate a VM. |
  - ### Uses of SimEvent in org.cloudbus.cloudsim.core

    Methods in org.cloudbus.cloudsim.core that return SimEvent

    | Modifier and Type | Method and Description |
    | --- | --- |
    |  |  |
    | --- | --- |
    | `static SimEvent` | CloudSim.`cancel(int src, Predicate p)` Removes an event from the event queue. |
    | `SimEvent` | SimEntity.`cancelEvent(Predicate p)` Cancel the first event matching a predicate waiting in the entity's future queue. |
    | `static SimEvent` | CloudSim.`findFirstDeferred(int src, Predicate p)` Find first deferred event matching a predicate. |
    | `protected SimEvent` | SimEntity.`getEventBuffer()` Gets the event buffer. |
    | `SimEvent` | SimEntity.`getNextEvent()` Get the first event waiting in the entity's deferred queue, or if there are none, wait for an event to arrive. |
    | `SimEvent` | SimEntity.`getNextEvent(Predicate p)` Get the first event matching a predicate from the deferred queue, or if none match, wait for a matching event to arrive. |
    | `static SimEvent` | CloudSim.`select(int src, Predicate p)` Selects an event matching a predicate. |
    | `SimEvent` | SimEntity.`selectEvent(Predicate p)` Extract the first event matching a predicate waiting in the entity's deferred queue. |

    Methods in org.cloudbus.cloudsim.core that return types with arguments of type SimEvent

    | Modifier and Type | Method and Description |
    | --- | --- |
    |  |  |
    | --- | --- |
    | `java.util.Iterator<SimEvent>` | DeferredQueue.`iterator()` Returns an iterator to the events in the queue. |
    | `java.util.Iterator<SimEvent>` | FutureQueue.`iterator()` Returns an iterator to the queue. |

    Methods in org.cloudbus.cloudsim.core with parameters of type SimEvent

    | Modifier and Type | Method and Description |
    | --- | --- |
    |  |  |
    | --- | --- |
    | `void` | DeferredQueue.`addEvent(SimEvent newEvent)` Adds a new event to the queue. |
    | `void` | FutureQueue.`addEvent(SimEvent newEvent)` Add a new event to the queue. |
    | `void` | FutureQueue.`addEventFirst(SimEvent newEvent)` Add a new event to the head of the queue. |
    | `int` | SimEvent.`compareTo(SimEvent event)` |
    | `void` | CloudInformationService.`processEvent(SimEvent ev)` Processes events scheduled for this entity. |
    | `void` | CloudSimShutdown.`processEvent(SimEvent ev)` The main method that shuts down hostList and Cloud Information Service (GIS). |
    | `abstract void` | SimEntity.`processEvent(SimEvent ev)` This method is invoked by the `Simulation` class whenever there is an event in the deferred queue, which needs to be processed by the entity. |
    | `protected void` | CloudInformationService.`processOtherEvent(SimEvent ev)` This method needs to override by a child class for processing other events. |
    | `boolean` | FutureQueue.`remove(SimEvent event)` Removes the event from the queue. |
    | `protected void` | SimEntity.`setEventBuffer(SimEvent e)` Sets the event buffer. |

    Method parameters in org.cloudbus.cloudsim.core with type arguments of type SimEvent

    | Modifier and Type | Method and Description |
    | --- | --- |
    |  |  |
    | --- | --- |
    | `boolean` | FutureQueue.`removeAll(java.util.Collection<SimEvent> events)` Removes all the events from the queue. |
  - ### Uses of SimEvent in org.cloudbus.cloudsim.core.predicates

    Methods in org.cloudbus.cloudsim.core.predicates with parameters of type SimEvent

    | Modifier and Type | Method and Description |
    | --- | --- |
    |  |  |
    | --- | --- |
    | `abstract boolean` | Predicate.`match(SimEvent event)` The match function which must be overridden when writing a new predicate. |
    | `boolean` | PredicateAny.`match(SimEvent ev)` The match function called by `Simulation`, not used directly by the user. |
    | `boolean` | PredicateFrom.`match(SimEvent ev)` The match function called by `Simulation`, not used directly by the user. |
    | `boolean` | PredicateNone.`match(SimEvent ev)` The match function called by `Simulation`, not used directly by the user. |
    | `boolean` | PredicateNotFrom.`match(SimEvent ev)` The match function called by `Simulation`, not used directly by the user. |
    | `boolean` | PredicateNotType.`match(SimEvent ev)` The match function called by `Simulation`, not used directly by the user. |
    | `boolean` | PredicateType.`match(SimEvent ev)` The match function called by `Sim_system`, not used directly by the user. |
  - ### Uses of SimEvent in org.cloudbus.cloudsim.examples

    Methods in org.cloudbus.cloudsim.examples with parameters of type SimEvent

    | Modifier and Type | Method and Description |
    | --- | --- |
    |  |  |
    | --- | --- |
    | `void` | CloudSimExample8.GlobalBroker.`processEvent(SimEvent ev)` |
  - ### Uses of SimEvent in org.cloudbus.cloudsim.network.datacenter

    Methods in org.cloudbus.cloudsim.network.datacenter with parameters of type SimEvent

    | Modifier and Type | Method and Description |
    | --- | --- |
    |  |  |
    | --- | --- |
    | `protected void` | NetDatacenterBroker.`processCloudletReturn(SimEvent ev)` Process a cloudlet return event. |
    | `protected void` | NetworkDatacenter.`processCloudletSubmit(SimEvent ev, boolean ack)` Processes a Cloudlet submission. |
    | `void` | NetDatacenterBroker.`processEvent(SimEvent ev)` Processes events available for this Broker. |
    | `void` | Switch.`processEvent(SimEvent ev)` |
    | `protected void` | Switch.`processhostpacket(SimEvent ev)` |
    | `protected void` | NetDatacenterBroker.`processOtherEvent(SimEvent ev)` Overrides this method when making a new and different type of Broker. |
    | `protected void` | AggregateSwitch.`processpacket_down(SimEvent ev)` Send Packet to switch connected through a downlink port |
    | `protected void` | Switch.`processpacket_down(SimEvent ev)` |
    | `protected void` | AggregateSwitch.`processpacket_up(SimEvent ev)` Send Packet to switch connected through a uplink port |
    | `protected void` | EdgeSwitch.`processpacket_up(SimEvent ev)` Send Packet to switch connected through a uplink port |
    | `protected void` | RootSwitch.`processpacket_up(SimEvent ev)` Send Packet to switch connected through a downlink port |
    | `protected void` | Switch.`processpacket_up(SimEvent ev)` |
    | `protected void` | Switch.`processpacket(SimEvent ev)` |
    | `protected void` | EdgeSwitch.`processpacketforward(SimEvent ev)` Send Packet to hosts connected to the switch |
    | `protected void` | Switch.`processpacketforward(SimEvent ev)` |
    | `protected void` | NetDatacenterBroker.`processResourceCharacteristics(SimEvent ev)` Process the return of a request for the characteristics of a PowerDatacenter. |
    | `protected void` | NetDatacenterBroker.`processResourceCharacteristicsRequest(SimEvent ev)` Process a request for the characteristics of a PowerDatacenter. |
  - ### Uses of SimEvent in org.cloudbus.cloudsim.power

    Methods in org.cloudbus.cloudsim.power with parameters of type SimEvent

    | Modifier and Type | Method and Description |
    | --- | --- |
    |  |  |
    | --- | --- |
    | `protected void` | PowerDatacenter.`processCloudletSubmit(SimEvent ev, boolean ack)` |
    | `protected void` | PowerDatacenterBroker.`processVmCreate(SimEvent ev)` |
    | `protected void` | PowerDatacenter.`processVmMigrate(SimEvent ev, boolean ack)` |
  - ### Uses of SimEvent in org.workflowsim

    Methods in org.workflowsim with parameters of type SimEvent

    | Modifier and Type | Method and Description |
    | --- | --- |
    |  |  |
    | --- | --- |
    | `protected void` | WorkflowScheduler.`processCloudletCheck(SimEvent ev)` process cloudlet (job) check (not supported yet) |
    | `protected void` | WorkflowScheduler.`processCloudletReturn(SimEvent ev)` Process a cloudlet (job) return event. |
    | `protected void` | WorkflowScheduler.`processCloudletSubmit(SimEvent ev)` Submits cloudlet (job) list |
    | `protected void` | DatacenterExtended.`processCloudletSubmit(SimEvent ev, boolean ack)` Processes a Cloudlet submission. |
    | `protected void` | WorkflowScheduler.`processCloudletUpdate(SimEvent ev)` Update a cloudlet (job) |
    | `void` | ClusteringEngine.`processEvent(SimEvent ev)` Processes events available for this Broker. |
    | `void` | WorkflowEngine.`processEvent(SimEvent ev)` Processes events available for this Broker. |
    | `void` | WorkflowPlanner.`processEvent(SimEvent ev)` Processes events available for this Broker. |
    | `void` | WorkflowScheduler.`processEvent(SimEvent ev)` Process an event |
    | `protected void` | WorkflowEngine.`processJobReturn(SimEvent ev)` Process a job return event. |
    | `protected void` | WorkflowEngine.`processJobSubmit(SimEvent ev)` Process a submit event |
    | `protected void` | ClusteringEngine.`processOtherEvent(SimEvent ev)` Overrides this method when making a new and different type of Broker. |
    | `protected void` | DatacenterExtended.`processOtherEvent(SimEvent ev)` |
    | `protected void` | WorkflowEngine.`processOtherEvent(SimEvent ev)` Overrides this method when making a new and different type of Broker. |
    | `protected void` | WorkflowPlanner.`processOtherEvent(SimEvent ev)` Overrides this method when making a new and different type of Broker. |
    | `protected void` | WorkflowEngine.`processResourceCharacteristicsRequest(SimEvent ev)` Process a request for the characteristics of a PowerDatacenter. |
    | `protected void` | WorkflowScheduler.`processResourceCharacteristicsRequest(SimEvent ev)` Process a request for the characteristics of a PowerDatacenter. |
    | `protected void` | WorkflowScheduler.`processVmCreate(SimEvent ev)` Process the ack received due to a request for VM creation. |

- Overview
- Package
- Class
- Use
- Tree
- Deprecated
- Index
- Help

- Prev
- Next

- Frames
- No Frames

- All Classes
